# Supplementary material for: Relative fat mass and cardiovascular risk in Peruvian adults: Findings from a national survey
Source: PLoS One. 2026 Feb 23;21(2):e0343062. doi: 10.1371/journal.pone.0343062 (PMC12928448; doi:10.1371/journal.pone.0343062)
Supplement: Attachment — Submitted filename: RESPONSE LETTER RFM FRSCORE.docx [file pone.0343062.s002.docx]

**RESPONSE LETTER**

*Authors’ comments: We thank both reviewers for their comments. With their input and the changes made, we believe that the manuscript has improved substantially.*

**Reviewer #1**:

The manuscript addresses an important public health question: the association between relative fat mass (RFM) and 10-year cardiovascular risk in a Peruvian population. The topic is timely, especially given the high burden of obesity and CVD in low- and middle-income countries. The paper is well-organized, methodologically sound, and provides valuable findings. However, several aspects require clarification and strengthening before the manuscript can be considered for publication.

**1.Novelty and Contribution**

**- The authors state this is the first study in Peru to link RFM with the Framingham risk score. While this is true, the novelty should be emphasized more clearly in the Introduction and Discussion. At present, the contribution is somewhat underplayed against the backdrop of existing international studies.**

***Response:*** *Thank you for the suggestion. We have added a text in the introduction acknowledging that differences in body composition and ethnicity modulate the association with cardiovascular risk*

***Changes****:* A text was added to the introduction section. Now it reads:

“Considering that differences in body composition and ethnicity modulate cardiovascular risk association [18], findings from high-income countries or predominantly Caucasian populations may not represent Latin American or Andean contexts.”

**2. Choice of Cardiovascular Risk Tool**

**- The Framingham risk score has not been validated in the Peruvian population, as the authors themselves note. This limitation is significant and may affect the accuracy of the estimates. The Discussion should more critically evaluate the implications of using this score and consider whether recalibrated regional risk equations (if available) might yield different results.**

***Response:*** *Thank you for the observation. We agree that lack of validation of the Framingham risk score in the Peruvian population may affect the accuracy of the estimates. This limitation was acknowledged in the Discussion section, where we state: “Although the Framingham score is widely used to estimate 10-year cardiovascular risk, its accuracy when applied outside the original U.S. population may lead to over- or underestimation of risk. In addition, the lack of validation in the Peruvian population may limit its applicability in this context.”*

*It is important to note that the Framingham risk score is endorsed by the Peruvian Ministry of Health as a reference tool for cardiovascular risk stratification, which supports its use in local clinical and epidemiological settings. We have incorporated an additional sentence acknowledging this.*

*Additionally, we have incorporated a text in the discussion section regarding the implications of this lack of validity in interpreting our findings*

***Changes:*** *In the discussion section we added the following text:*

“However, our results should be interpreted with caution as the Framingham risk score has neither been validated nor recalibrated for the Peruvian population. Chile is one of the few countries in the region that has undertaken efforts to recalibrate the model for its own population [37]. Since the score was originally developed using baseline cardiovascular event rates from a U.S. cohort, the absolute levels of predicted 10-year risk may differ substantially from those in the Peruvian context and other Andean countries. Nevertheless, the model likely preserves the relative ranking of individuals indicating who presents higher or lower estimated cardiovascular risk.”

*We have added* *the following text to the limitations section:*

“Nevertheless, the score is currently recommended by the Peruvian Ministry of Health for cardiovascular risk stratification [16], which supports its use in local epidemiological analyses”

**3. Cross-Sectional Design**

**- The limitation that causality cannot be inferred should be highlighted earlier (e.g., in the Abstract or Introduction), not only in the Discussion. The authors should also consider whether reverse causality (e.g., CVD risk factors influencing body fat distribution) could bias results.**

***Response:*** *Thank you for this observation. According to the STROBE report guidelines (https://pmc.ncbi.nlm.nih.gov/articles/PMC2034723/), the study limitations should be reported in the Discussion section. They recommend summarizing what was done and found in the Abstract, while the Introduction should present the state of the art regarding the problem and outline the objectives. In the Discussion section, the limitations of the study should indeed be addressed, as specified in the guideline. Therefore, in order to follow established reporting standards, we have kept the limitations in the Discussion section.*

*Regarding the possibility of reverse causality, we consider it unlikely in this context. The Framingham Risk Score represents an estimate of future cardiovascular risk rather than past or current clinical event. Thus, it cannot temporally precede or influence current body composition. For this reason, due to the nature of the Framingham Risk Score, reverse causality with the RFM—which does estimate current body fat percentage—is very unlikely. Additionally, we consider that having already stated in the abstract that our study will assess an association is sufficient for the reader’s understanding.*

***Changes:* None**

**4. Definition of Obesity via RFM**

**- The rationale for the chosen cut-off points (≥40% for women and ≥30% for men) is based on international validation studies. However, whether these cutoffs are appropriate for the Peruvian population is uncertain. Could the authors provide sensitivity analyses using alternative thresholds or justify more strongly why these cutoffs are optimal locally?**

***Response:*** *Thank you for the observation. We agree on the importance of the cut-off selection for defining obesity using the RFM. Nevertheless, as ethnic and anthropometric diversity across countries can influence body composition, cut-off points developed in other populations may not be appropriate for the Peruvian context. In line with this, the Lancet Commission on clinical obesity recommends using country-specific cut-off points when determining an obesity diagnosis (1). However, given the absence of validated RFM cut-off points for the Peruvian population and the variability of those proposed in different countries (Table A), we decided to use the original study’s cut-offs (≥40% for women and ≥30% for men). It is important to note that to address concerns regarding threshold dependence, we also conducted a sensitivity analysis treating RFM as a continuous variable, without applying a specific cut-off.*

1. Rubino F, Cummings DE, Eckel RH, et al. Definition and diagnostic criteria of clinical obesity. Lancet Diabetes Endocrinol. 2025;13(3):221-262. doi:10.1016/S2213-8587(24)00316-4

| **Table A. Cutoff points for defining obesity according to RFM used in different countries.** | | |
| --- | --- | --- |
| **Study** | **Population study** | **Cut-off-point** |
| ***Usefulness of relative fat mass in estimating body adiposity in Korean adult population***  [**https://www.jstage.jst.go.jp/article/endocrj/66/8/66_EJ19-0064/_article/-char/ja/**](https://www.jstage.jst.go.jp/article/endocrj/66/8/66_EJ19-0064/_article/-char/ja/) | Corea | ≥25 men  ≥35 women |
| ***Relative fat mass and prediction of incident atrial fibrillation, heart failure and coronary artery disease in the general population***  [**https://www.nature.com/articles/s41366-023-01380-8**](https://www.nature.com/articles/s41366-023-01380-8) | Netherlands | ≥26 men  ≥38 women |
| ***Predictive values of relative fat mass and body mass index on cardiovascular health in community-dwelling older adults: Results from the Longevity Check-up (Lookup) 7+***  [**https://www.maturitas.org/article/S0378-5122(24)00106-3/fulltext**](https://www.maturitas.org/article/S0378-5122(24)00106-3/fulltext) | Italy | ≥27 men  ≥40 women |
| ***Relative fat mass as an estimator of body fat percentage in Chilean adults***  [**https://www.nature.com/articles/s41430-024-01464-2**](https://www.nature.com/articles/s41430-024-01464-2) | Chile | ≥22,7 men  ≥32,4 women |

*In addition, and in response to the reviewer’s concern, we conducted an additional sensitivity analysis using a statistical criterion based on tertiles and is reported in the New Table 3. The results showed the same pattern as those obtained with the original cut-offs.*

***Changes:*** *We conducted an additional sensitivity analysis using a tertile-based classification derived from our own sample. The results of this analysis have been incorporated into the revised manuscript and added to Table 3.*

| **Table 3.** Associations between Relative Fat Mass and the 10-year Framingham Risk Score by sex | | | | | |
| --- | --- | --- | --- | --- | --- |
|  | **Generalized linear model (GLM) of the gamma family** | | | | |
|  | **Crude** |  |  | **Adjusted*** |  |
| **Model** | **β (95% CIs)** | **SE** |  | **β (95% CIs)** | **SE** |
| **Male** |  |  |  |  |  |
| Obesity by Relative Fat Mass |  |  |  |  |  |
| No | Reference |  |  | Reference |  |
| Yes | 0.59 (0.37-0.81)^~~a~~^ | 0.11 |  | 0.39 (0.23-0.56)^~~b~~^ | 0.08 |
| **Female** |  |  |  |  |  |
| Obesity by Relative Fat Mass |  |  |  |  |  |
| No | Reference |  |  | Reference |  |
| Yes | 0.66 (0.42-0.91)^~~c~~^ | 0.12 |  | 0.4~~7~~8 (0.32-0.63)^~~d~~^ | 0.08 |
| **Male** |  |  |  |  |  |
| Tertiles by Relative Fat Mass |  |  |  |  |  |
| Low | Reference |  |  | Reference |  |
| Medium | 0.57 (0.30-0.84) | 0.14 |  | 0.39 (0.19-0.59) | 0.10 |
| High | 0.83 (0.56-1.09) | 0.13 |  | 0.61 (0.42-0.80) | 0.10 |
| **Female** |  |  |  |  |  |
| Tertiles by Relative Fat Mass |  |  |  |  |  |
| Low | Reference |  |  | Reference |  |
| Medium | 0.32 (0.05-0.60) | 0.14 |  | 0.19 (0.03-0.35) | 0.08 |
| High | 0.68 (0.41-0.95) | 0.14 |  | 0.47 (0.28-0.67) | 0.10 |
| **Male** |  |  |  |  |  |
| Relative Fat Mass | 0.10 (0.07-0.12) | 0.01 |  | 0.07 (0.05-0.09) | 0.01 |
| **Female** |  |  |  |  |  |
| Relative Fat Mass | 0.06 (0.03-0.08) | 0.01 |  | 0.06 (0.04-0.08) | 0.01 |

SE: standard error, CIs: confidence intervals, β: coefficient. All estimates accounted for the VIANEV sample design. * Adjusted for age group, area of residence, educational level, sitting time, poverty status, fruit and vegetable intake, alcohol consumption and altitude of residence.

**5. Statistical Methods**

**-The use of generalized linear models of the gamma family with a log link is appropriate for skewed outcomes. However, the paper would benefit from an explanation of why this model was selected over alternatives (e.g., quantile regression). Additionally, please clarify whether survey weights and clustering were fully accounted for in all regression analyses.**

***Response:*** *Thank you for the recommendation. We agree that several modeling strategies may be used for skewed outcomes. However, our research question focused on estimating the association between RFM and the Framingham Risk Score. For this purpose, a generalized linear model with a gamma family and log link was selected because it models the conditional mean of a positively skewed continuous outcome and provides multiplicative, population-averaged estimates, which are appropriate for addressing this type of association. Quantile regression, while valuable for examining how an exposure affects specific quantiles of the outcome distribution, addresses a different scientific question and is not designed to estimate mean associations. Because our study did not aim to investigate distributional differences across percentiles, quantile regression was not the optimal analytic approach. Additionally, for quantile regression STATA statistical package does not account for the complex survey weights. This would yield imprecise estimates given the national survey we used, and thus represents a limitation.*

*Regarding the use of survey weights and the complex survey design in regression models we would like to note that in the submitted manuscript we declared in the Statistical Analysis section that “Given the characteristics of the VIANEV survey, the analyses accounted for sample weights and the complex survey design”. Furthermore, for further clarity Table 3 has a footnote stating this, and it reads “All estimates accounted for the VIANEV sample design”*

***Changes****: None*

**6. Potential Confounders**

**-Important confounders such as alcohol consumption, physical activity beyond sedentary time, and dietary quality were not included. The absence of these variables should be acknowledged as a limitation in more detail.**

***Response:*** *Thank you for the observation. When selecting potential confounding variables for our models, we considered including alcohol consumption, which is available in the dataset (alcohol use during the past 30 days). Initially, we decided not to include it because our primary focus was on variables most directly related to adiposity and cardiometabolic risk; however, we agree that alcohol consumption is an important behavioral factor. Based on your suggestion, we have now incorporated alcohol consumption into all adjusted models (see revised Tables 1, 2, and 3). After inclusion, the coefficients remained virtually unchanged, with only one estimate showing a minimal increase (from 0.47 [0.32–0.63] to 0.48 [0.32–0.63]), indicating that alcohol consumption did not materially alter the associations.*

*Regarding physical activity, we opted to adjust for sedentary time because prolonged sitting is independently associated with adverse cardiometabolic outcomes, even among individuals who meet physical activity recommendations (1). Sedentary behavior therefore captures a distinct and relevant dimension of movement-related exposure. Nonetheless, we acknowledge that the absence of more detailed physical activity indicators may result in residual confounding. No dietary quality data was available for which we did not include this as a confounding variable.*

*1. Liang, Zhi-de et al. “Association between sedentary behavior, physical activity, and cardiovascular disease-related outcomes in adults-A meta-analysis and systematic review.” Frontiers in public health vol. 10 1018460. 19 Oct. 2022, doi:10.3389/fpubh.2022.1018460*

***Changes:*** *Following your recommendation, we have added the following to our limitations section:*

“The lack of potentially confounding variables, such as diet quality, energy intake or more specific physical activity measurement, could also result in residual confounding, affecting the magnitude and direction of the estimated associations due to its strong influence on body fat percentage and components of the Framingham Risk Score; nevertheless, the adjustment models included indirect measures of diet quality (daily consumption of five or more portions of fruits and vegetables) and physical activity (daily hours of sedentary behavior) in order to reduce confounding.”

**7. Sex-Specific Findings**

**- The stronger association in women is intriguing. The Discussion offers some explanations (biological and social), but these remain speculative. The authors should expand on possible mechanisms and highlight the need for future longitudinal research to clarify causality.**

***Response:*** *Thank you for the observation. We agree with the reviewer’s observation and have incorporated a text addressing this topic.*

***Changes:*** *The following text was added in the discussion section:*

“Additionally, our findings show a stronger association between obesity and cardiovascular risk in women, which is consistent with evidence indicating a higher burden of cardiovascular risk factors in women compared to men [38]. In Peru, studies have similarly reported a higher prevalence of obesity and metabolic syndrome among women [39,40]. Likewise, an analysis of the nationally representative ENDES 2019 survey found that women had markedly higher odds of abdominal obesity than men, reinforcing the notion of sex-specific vulnerability in adiposity-related cardiometabolic risk [40]. Together, these findings provide context for why increases in RFM may correspond to larger relative changes in predicted 10-year cardiovascular risk among women in our study.

~~In line with this, biological factors, such as greater susceptibility to chronic diseases and the interaction between estrogens and age, as well as social factors including disparities in the detection and treatment of CVD, could also explain women’s higher vulnerability [38].~~ On the other hand, epidemiological studies in Latin America [4] have reported a higher incidence of CVD in men than in women (655 vs. 531 per 100,000 individuals), and additional evidence indicates that Peruvian men present worse cardiovascular health than women [5,41]. Of note, compared with previous reports, our results address related but not identical dimensions of cardiovascular risk. In this line, evidence from Colombia has shown that although women presented a higher prevalence of several cardiovascular risk factors, including obesity, hypertension, and dyslipidemia, the incidence of major cardiovascular events over 12 years was substantially higher in men [42]. This pattern suggests that a greater burden of risk factors does not necessarily translate into higher event incidence, and that sex-specific patterns of predicted risk may not align perfectly with observed outcomes. Importantly, these observations do not contradict our results. The Framingham Risk Score estimates predicted 10-year cardiovascular risk based on an algorithm incorporating multiple components (blood pressure, lipid profile, smoking, diabetes, age, and sex). Consequently, variation in these components, independent of the observed incidence of cardiovascular events, modifies the estimated risk. While we assessed predicted 10-year risk using the Framingham score, previous studies reported observed incidence of cardiovascular events. These perspectives are complementary and highlight the need to consider both estimated risk and actual incidence when interpreting sex-specific patterns. This distinction underscores the need for longitudinal studies in Peru to determine whether adiposity exerts differential cardiometabolic effects by sex and how these differences translate into actual cardiovascular outcome.”

**8. Abstract**

**- The Abstract is concise, but the phrase “positively associated” could be replaced with a more precise quantitative description (e.g., “associated with a 61% higher predicted risk in women”).**

***Response:*** *Because we have decided to exclude the PRs for statistical clarity (see our response to your comment number 11), we will keep this statement in the abstract and will not make any modifications. Moreover, given our study design, we cannot claim a ‘predicted risk,’ as we only established an association. Nevertheless, we appreciate your observation.*

***Changes:*** *None*

**9. Line 63–69:**

**The claim that RFM’s discriminatory capacity surpasses BMI and waist circumference should be better referenced, and the cited evidence summarized more explicitly.**

***Response:*** *Thank you for the observation. The sentence was revised to clarify the references used. In addition, we included one more result from the cited study.*

***Changes:*** *The referred text now reads:*

“Moreover, its predictive discrimination for heart disease mortality (0.64 in women and 0.67 in men) has been shown to be higher than that of body mass index (0.53 and 0.56) and waist circumference (0.60 and 0.64) [12] In addition, compared to body mass index (BMI) and waist circumference, RFM has demonstrated a greater predictive discrimination for heart disease mortality in both sexes. Likewise, the proportion of preventable heart disease mortality attributed to RFM-diagnosed obesity was higher than that observed when using BMI or WC [12]. These findings add to previous evidence that also supports the use of RFM as a useful tool for predicting cardiovascular risk [13].”

**10. Methods**

**- Please specify how missing data were handled (listwise deletion vs. imputation).**

***Response:*** *Thank you for the observation. In the Study Population section, we stated that participants with missing values in any of the variables of interest were excluded.*

***Changes:*** *None*

**- Clarify whether waist circumference was measured at the midpoint between the lowest rib and iliac crest or at another anatomical site.**

***Response:*** *Thank you for the observation. We have incorporated the information requested*

***Changes:*** *The following was added:*

“Obesity was defined using the RFM index, calculated from height (cm), waist circumference (cm), and sex of participants. Waist circumference was measured using a 200-cm measuring tape with 1-mm precision, positioning it at the midpoint between the lower margin of the last rib and the upper border of the iliac crest. Height was assessed with a fixed wooden stadiometer [20]. The following calculation was performed [11]:”

**11. Results**

**- Tables are informative but could be more reader-friendly if prevalence ratios (PRs) were included directly in Table 3 rather than only in the footnotes.**

***Response:*** *Thank you for the suggestion. While we agree that the PRs could be interpreted more easily, it is important to note that all the models we used have a quantitative outcome, and therefore the gamma GLM estimates coefficients. Therefore, we consider that, for statistical clarity, we will keep only the coefficients and will proceed to remove the PRs from the table.*

***Changes:*** *None*

**- Consider reporting absolute mean differences in Framingham scores between obese and non-obese participants for interpretability.**

***Response:*** *Thank you for the recommendation. We have included the mean differences in the Framingham Score for men and women according to obesity status based on RFM.*

***Changes:*** *Mean differences in Framingham scores between obese and non-obese within each sex was calculated and reported. The added text reads:*

“Additionally, when comparing the Framingham Score between non-obese and obese participants, non-obese men (7.65) had a lower mean Framingham Score than obese men (13.75) (mean difference: –6.11, 95% CI: –8.63 to –3.58, p < 0.001). Similarly, non-obese women (2.07) had a lower Framingham Score than obese women (4.01) (mean difference: –1.95, 95% CIs: –2.63 to –1.27, p < 0.001).”

**12. Discussion**

**- The section on public health implications (lines 263–280) could be expanded to comment on how RFM might realistically be integrated into Peruvian clinical practice and whether it could replace or complement BMI in national guidelines.**

***Response:*** *Thank you for the recommendation. We have expanded the implications on the discussion section*

***Changes:*** *The following was added:*

“These findings seek to contribute to the growing body of evidence supporting the need to implement alternative diagnostic criteria for obesity that can more accurately estimate cardiometabolic risk in Peru. To advance this effort, the main step is to draw on the experience of countries such as the United States [49] and the United Kingdom [50]—where WC and WHtR measurements are endorsed by clinical guidelines for cardiovascular risk assessment—and to establish RFM cut-off-points for the Peruvian population, given the country’s distinct ethnic characteristics. This requires population-based studies that include a valid reference standard for adiposity, such as DEXA, as well as cardiometabolic outcomes. Moreover, an important advancement in Peru’s primary care system is the normative use of waist circumference. Therefore, the implementation of other anthropometric indicators that also rely on this measurement could be facilitated and strengthened.”

**- Please update references to regional guidelines or ongoing WHO/PAHO initiatives to strengthen applicability.**

***Response:*** *Thank you for the recommendation. We have updated references on this topic as suggested.*

***Changes:*** *The following was added:*

“In the Region of the Americas, particularly in low- and middle-income countries such as Peru, CVD remains one of the leading causes of mortality. In response, and within the framework of the Global Action Plan for the Prevention and Control of Noncommunicable Diseases (NCDs) [43], the World Health Organization has promoted the Global HEARTS Initiative [44], and its regional adaptation, HEARTS in the Americas, which aims to strengthen health service performance for CVD prevention and control.”

**Reviewer #2:**

well structured manuscipt. although, stduy have not add a new to knowledge as the topic is previousely known, the following suggestions may improve it more :

**Methodology:**

**1. because you retrieve a seconday data so its preferde to refered as Retreospective SURVEY correation design)**

***Response:*** *Thank you for the suggestion. We appreciate the intention behind the term “retrospective”; however, incorporating it into the description of our design may lead to conceptual ambiguity. According to the “A Dictionary of Epidemiology, Sixth Edition” (1), “retrospective” is typically used in the context of evaluating etiological hypotheses by looking back at past exposures, a usage that is strongly associated with specific analytical study designs. Although our study uses previously collected data, our aim was not to test causal hypotheses but to analyze associations within an existing dataset. For this reason, labeling the design as “retrospective” may unintentionally suggest methodological features that do not apply to our study.*

*1. Porta, M., Greenland, S., Hernán, M., dos Santos Silva, I., & Last, J. M. (Eds.). (2014). A Dictionary of Epidemiology (6ª ed.). Oxford University Press.*

*Regarding the use of the term ‘Correlational,’ we have decided not to include it, as no correlation analysis was performed.* ***A more detailed explanation can be found in our response to your comment number 6, where you suggested performing correlation analyses.***

***Changes:*** *None*

**2. * explain the sampling processes in you maunscript (*how you select the participants within your study) as what discussed here is about the population and inclusion criteria of primary survey!**

***Response:*** *Thank you for the suggestion. We have carefully reviewed our manuscript, and this has already been mentioned. Information on how we selected the participants for our study can be found in the Study Population section , and details on the population and inclusion criteria of the primary survey are provided in the Study Type and Design section.*

***Changes:*** *None*

**3. * most important is regaerding the Framingham risk score : WHEN AND HOW THE STUDY WAS APPLIED THE SCORE ? ; the scoring system must be also eplained**

***Response:*** *Initially, we did not provide extensive details about the Framingham Heart Study because it is a well-known study. Therefore, we only cited the original reference and indicated the procedure used for its calculation in the statistical software (Stata). To provide additional information about the study, we have now included the following details:*

***Changes:*** *The following was added:*

“The 10-year cardiovascular risk was analyzed as a continuous variable and estimated using the Framingham risk score [14]. This score was proposed by D’Agostino et al. in 2008 using data from adults enrolled in the Framingham Heart Study. For the development of this score, eligible participants were those who attended examination cycles conducted between 1968 and 1987, had high-density lipoprotein cholesterol measurements available, and were free of cardiovascular disease at baseline. From the initial assessment, the incidence of cardiovascular events was monitored, and the risk factors that significantly predicted these events were evaluated to construct a points-based score for estimating 10-year cardiovascular risk. The score assigns risk points through an algorithm that incorporates previously identified factors: age (years), sex, systolic blood pressure, high-density lipoprotein cholesterol, total cholesterol, smoking status, diabetes diagnosis (defined as fasting glucose ≥126 mg/dL or use of diabetes-related medication), and use of antihypertensive medication. Higher scores indicate greater risk of cardiovascular events within 10 years [14]. The score was calculated using the *framingham* command in Stata SE version 18.”

**4. confusion was arised between the VIANEV and ENAHP ?? explain!**

***Response:*** *Thank you for the observation. We have re-written the referred text for further clarity.*

***Changes:*** *The following was added:*

~~“The VIANEV 2017–2018 sampling frame was based on households included in the National Household Survey (ENAHO), one of the largest population-based surveys in Peru and widely used to inform health decision-making and guide public policies in the country~~ The VIANEV 2017–2018 survey was conducted among a subsample of adults who also participated in the National Household Survey 2017 (ENAHO). ENAHO constitutes one of the largest population-based surveys in Peru and widely used to inform health decision-making and guide public policy in the country. In contrast to ENAHO, which focuses primarily on demographic and economic aspects, VIANEV participants were asked a broader set of health-related questions.”

**5. statistical analysis: median is not required as the RFM is a continous variable (Mean is enough)**

***Response:*** *Thank you for the suggestion. It is important to clarify the following: to address the objective of our article, we used an association GLMs from the gamma family with a logarithmic link function. To apply this model, our outcome variable (Framingham Risk Score) must be skewed (right-skewed), indicating a non-normal distribution. In such cases, the appropriate measure of central tendency is the median, which we have presented in Table 2. Nevertheless, quantitative variables with large numbers of observations are often assumed to approximate a normal distribution. Given that our analysis is based on a survey with sampling weights and a substantial sample size, we decided to additionally report the mean so as not to restrict the interpretation to a single measure.*

*For this reason, we consider it appropriate to retain both measures of central tendency*

***Changes:*** *None*

**6. correlation coefficient is required to investigate the correlation**

***Response:*** *Our main objective was to evaluate the association between RFM (both as a dichotomous and continuous variable) and the Framingham Risk Score (continuous variable). Therefore, we used an association of GLMs from the gamma family with a logarithmic link function. This model provides an estimation that allows us to accomplish three main objectives: 1) quantify the strength/magnitude of the association to understand the change in cardiovascular risk according to the change in RFM (modeling); 2) determine the direction of the association (positive or negative); and 3) adjust for potential covariates that could confound the association between RFM and cardiovascular risk, yielding a more precise estimate.*

*On the other hand, calculating a correlation coefficient between our variables using Spearman’s correlation test (suitable for numerical variables with a non-normal distribution, as in our case) would only provide a coefficient indicating a bivariate positive or negative association. This coefficient is limited for the purposes of this study because its units do not reflect the measurement of our variables.*

*For these reasons, given the limitations of correlation tests and the benefits of the association model employed, we determined that performing correlation analyses would not be the most appropriate approach for our objectives.*

***Changes:*** *None*

**7. Results : MASL abberivation (NO space )**

***Response:*** *We appreciate your observation. It has been revised.*

***Changes:*** *We have made the corrections accordingly.*

**8. TABLE 2; you have to include the correlation coefficient (r) and sig so the table must provide a valuable data**

***Response:*** *Based on the response and justification provided to your comment number 6, we have decided not to include correlation coefficients in our study.*

***Changes:*** *None*

**9. table 3 ; full detailed regression ,odel must be provided so the study can predicte or exclude the cofounding factors**

***Response:*** *Thank you for the recommendation.*

*We understand that the purpose of your suggestion is to provide detailed information on which confounding variables were excluded from the adjusted model and to state the criteria used for this. In our study, we decided to perform adjustment based on an epidemiological criterion rather than a statistical criterion. The epidemiological criterion helped us identify potential confounding variables and include all of them in the adjusted models. Previously, in the covariates section, we had mentioned that all covariates were selected based on their epidemiological relevance.*

*We considered it necessary to clarify this further; therefore, we have added an additional sentence in the Statistical Analysis section specifying that all adjustment variables included in the model were those declared as potential confounding variables in the covariates section.*

***Changes:*** *The following was added:*

“To assess the association between RFM and the Framingham risk score, crude and adjusted generalized linear models (GLMs) from the gamma family with a logarithmic link function were employed. All covariates were included in the adjusted models based on their epidemiological relevance. This model was chosen because it is appropriate for positively skewed continuous outcomes such as Framingham score values.”

**10. duiscussion: well, its preffered to also add discussion about the table 2 (after you made the mentioned comments regarding the tabel 2)**

**Tampoco.**

***Response:*** Thank you for the recommendation. However, since correlation analyses will not be included, as justified in our response to your comment number 6, this change will not be necessary.

***Changes:*** *None*

**11. conclusion: must include the main finding of table 2**

***Response:*** Thank you for the recommendation. However, Since correlation analyses will not be included, as justified in our response to your comment number 6, this change will not be necessary.

***Changes:*** *None*
