# Supplementary material for: Relative fat mass and cardiovascular risk in Peruvian adults: Findings from a national survey
Source: PLoS One. 2026 Feb 23;21(2):e0343062. doi: 10.1371/journal.pone.0343062 (PMC12928448; doi:10.1371/journal.pone.0343062)
Supplement: Attachment — Submitted filename: 1. Response Letter R2.docx [file pone.0343062.s003.docx]

**Response Letter - Round 2**

**Reviewer #1:**

**1. Novelty and Framing of Contribution**

**Although the authors now acknowledge ethnic and body composition differences in the Introduction, the unique contribution of this study remains under-emphasized.**

**- The manuscript would benefit from a clearer statement of contribution beyond “first in Peru.”**

**- Specifically, the authors should emphasize:**

**- The comparison of multiple operationalizations of RFM (binary, continuous, tertiles)**

**- The sex-stratified modeling with complex survey adjustment**

**Recommendation:**

**Add 1–2 sentences in the final paragraph of the Introduction explicitly stating how this study advances knowledge beyond prior international RFM studies.**

**Response:**

We thank the reviewer for this important observation. We agree that our contribution extends beyond being the first study in Peru and should highlight the methodological advances and analytical approach that distinguish our work from previous international studies. We have revised the final paragraph of the Introduction to better articulate these specific contributions.

**Change:**

Now the text reads: Therefore, this study aimed to evaluate the association between obesity defined by RFM, considered under different operationalization approaches (binary, continuous, and tertile-based) and 10-year cardiovascular risk stratified by sex, as estimated by the Framingham score in Peruvian adults who participated in a national survey during 2017–2018. The present study advances knowledge beyond prior international RFM studies by comparing multiple operationalizations of RFM (binary, continuous, and tertiles) and applying sex-stratified analyses within a complex survey design in a nationally representative Latin American population, providing novel insights into optimal analytical strategies for RFM-cardiovascular risk assessment in ethnically diverse contexts.

**2. Use of the Framingham Risk Score**

**The authors appropriately acknowledge the lack of local validation and justify the use of the Framingham score due to Ministry of Health endorsement. However:**

**- The manuscript still risks being interpreted as estimating true cardiovascular risk rather than relative predicted risk.**

**- Some phrasing in the Results and Discussion (e.g., “higher cardiovascular risk”) could be misinterpreted by readers unfamiliar with risk score limitations.**

**Recommendation:**

**Systematically use wording such as “higher estimated 10-year cardiovascular risk” or “higher Framingham risk score” throughout the Results and Discussion to avoid overinterpretation.**

**Response**:

We thank the reviewer for this observation. We fully agree that our terminology must clearly distinguish between predicted risk scores and actual cardiovascular risk, especially given the lack of local validation of the Framingham score in Peruvian populations.

**Change:**

We have made changes to the Results and Discussion sections to explicitly specify that the outcome refers to “risk.”

**3. Interpretation of Effect Sizes from Gamma GLM**

**The use of β coefficients from a log-linked gamma model is statistically sound, but interpretability remains limited for non-technical readers.**

**- While the authors chose not to report prevalence ratios, readers may still struggle to understand the clinical relevance of coefficients such as β = 0.48.**

**- The newly added absolute mean differences are helpful but underutilized.**

**Recommendation:**

**In the Discussion, briefly interpret at least one adjusted estimate in plain language (e.g., relative or proportional increase in Framingham score) to improve accessibility.**

**Response:**

We thank the reviewer for this observation. We agree with the suggestion and have added a plain-language interpretation in the first paragraph of the Discussion section.

**Change:**

The objective of the present study was to evaluate the association between obesity defined by RFM and the estimated 10-year cardiovascular risk according to ~~estimated through~~ the Framingham risk score in Peruvian adults aged 30 to 59 years. A positive and significant association was observed in both sexes. Furthermore, it was found that for each unit increase in RFM, the estimated cardiovascular risk ~~Framingham risk score~~ increased by 0.07 in men and 0.06 in women, corresponding to approximately 7% and 6% higher estimated risk, respectively. This indicates that higher estimated total body fat percentage is associated with proportionally higher estimated 10-year cardiovascular risk ~~indicating that for each increase in the estimated total body fat percentage, the estimated 10-year cardiovascular risk also increases.~~ To the best of our knowledge, this is the first study to evaluate the association between RFM, an alternative estimator of total body fat percentage, and the estimated 10-year cardiovascular risk using the Framingham ~~risk~~ score.

**4. Sex-Specific Findings**

**The expanded discussion on sex differences is thoughtful and well-referenced. However:**

**- The section is now quite long and diffuse, mixing Peruvian data, regional incidence, and methodological distinctions between predicted risk and observed events.**

**- Some arguments appear defensive rather than explanatory.**

**Recommendation:**

**Condense this section slightly and clearly separate:**

**1.Biological/social explanations**

**2.Differences between predicted risk and observed incidence**

**3.Implications for future longitudinal research**

**This will improve coherence and readability.**

**Response:**

We thank the reviewer for this observation. We have thoroughly reviewed the content of the Discussion section, which was was refined and reorganized to improve coherence and readability with added subheadings to facilitate identification of the following sections:

1) Comparison with previous studies

2) Potential explanatory mechanisms

3) Sex-based differences

4) Implications for public health and clinical practice

5) Study limitations.

**Changes:** The discussion section has been revised. The sex-specific findings section was refined and reorganized to improve clarity.
