# Supplementary material for: Relative fat mass and cardiovascular risk in Peruvian adults: Findings from a national survey
Source: PLoS One. 2026 Feb 23;21(2):e0343062. doi: 10.1371/journal.pone.0343062 (PMC12928448; doi:10.1371/journal.pone.0343062)
Supplement: Attachment — Submitted filename: Response Letter R3.docx [file pone.0343062.s004.docx]

**Response Letter - Round 3**

**Reviewer #1:**

**1. Clarification of Outcome Interpretation**

**The authors have appropriately revised much of the text to emphasize “estimated 10-year cardiovascular risk” rather than true event risk. However, a small number of phrases still risk causal or clinical overinterpretation, particularly in the Abstract and Conclusion where statements such as “higher cardiovascular risk” appear without explicit reference to prediction.**

**Recommendation:**

**- Perform a final consistency check across the Abstract, Results, Discussion, and Conclusion to ensure uniform use of terms such as “higher Framingham risk score” or “higher estimated 10-year cardiovascular risk.”**

**This will further reduce the risk of misinterpretation by non-specialist readers.**

**Response:** We thank the reviewer for this observation.

**Changes:** Revisions have been made throughout the manuscript in accordance with the recommendations.

**2. Interpretation of Effect Sizes in Log-Gamma Models**

**The added plain-language interpretation of the continuous RFM coefficients (e.g., ~6–7% higher estimated risk per unit increase) is a valuable improvement. However, the interpretation currently appears only once and is not clearly tied to the categorical (binary and tertile-based) analyses.**

**Recommendation:**

**- Briefly contextualize at least one categorical comparison (e.g., obese vs. non-obese or highest vs. lowest tertile) in relative or proportional terms in the Discussion.**

**This would enhance accessibility for readers unfamiliar with generalized linear models while preserving statistical rigor.**

**Response:** Thank you for the clarification. We have incorporated these recommendations into the Discussion section.

**Changes:** We added interpretations in proportional terms, as well as interpretations of the results stratified by tertiles.

**3. Cross-sectional Design and Temporality**

**Although the limitations section appropriately acknowledges the cross-sectional nature of the data, some statements in the Discussion (particularly in the mechanistic and public health implications sections) still imply directionality between adiposity and cardiovascular risk.**

**Recommendation:**

**- Slightly temper causal language when referring to mechanisms or implications (e.g., “may contribute to” rather than “leads to”).**

**- Explicitly reiterate that associations reflect concurrent relationships with predicted risk rather than disease progression.**

**Response:** We thank the reviewer for this observation. We revised the Discussion to further temper causal language, particularly in the sections addressing potential mechanisms and public health implications.

**Changes**: Revisions have been made throughout the manuscript in accordance with the recommendations. Relevant statements were rephrased to emphasize that our findings reflect concurrent associations between RFM-defined adiposity and estimated 10-year cardiovascular risk, rather than causal effects or disease progression.

**4. Sex-Stratified Analyses**

**The reorganization of the Discussion into sub-sections has improved clarity. The section on sex-based differences is now more balanced and less defensive. However, it remains relatively long compared to other sections.**

**Recommendation:**

**- Consider further condensation by reducing repetition between regional epidemiology and methodological explanations of predicted versus observed risk.**

**Response:** Thank you for the clarification. We agree that this section is relatively lengthy.

**Change:** We have synthesized the main ideas of this subsection while preserving the key message we aim to convey to readers.

**5. Covariate Selection and Residual Confounding**

**The rationale for covariate selection is reasonable and consistent with epidemiological standards. However, diet and physical activity are only indirectly captured.**

**Recommendation:**

**- Add a brief sentence in the Methods or Limitations explicitly stating that residual confounding related to unmeasured dietary quality and physical activity intensity cannot be excluded.**

**Response**: Thank you for the clarification. We agree that it is important to explicitly state that adjustment for these variables does not exclude residual confounding.

**Changes**: We have clarified this in the Limitations section.

**6. Presentation of Tables**

**Tables are generally clear and informative. However, Table 2 is dense and may be difficult to interpret for readers.**

**Recommendation:**

**- Consider adding a brief interpretive sentence in the Results highlighting the most salient gradients (e.g., age and altitude trends) rather than relying solely on tabular detail.**

**Response:** Thank you for the clarification regarding synthesizing the findings of Table 2 in the Results section.

**Changes:** We have made changes to the interpretation of the findings.
